# Clinical effects of transumbilical laparoendoscopic single-site extracorporeal surgery in treatment of giant ovarian cysts and huge cystic pelvic masses

**DOI:** 10.12669/pjms.41.8.11514

**Published:** 2025-08

**Authors:** Yanqiong Hu, Yanyan Wang, Binzhou Sun, Rui Li, Luqian Ge

**Affiliations:** 1 Yanqiong Hu Department of Gynaecology, Maternity & Child Care Center of Qinhuangdao, Qinhuangdao 066000, Hebei, China; 2 Yanyan Wang Department of Gynaecology, Maternity & Child Care Center of Qinhuangdao, Qinhuangdao 066000, Hebei, China; 3 Binzhou Sun Department of Gynaecology, Maternity & Child Care Center of Qinhuangdao, Qinhuangdao 066000, Hebei, China; 4 Rui Li Department of Gynaecology, Maternity & Child Care Center of Qinhuangdao, Qinhuangdao 066000, Hebei, China; 5 Luqian Ge Department of Gynaecology, Maternity & Child Care Center of Qinhuangdao, Qinhuangdao 066000, Hebei, China

**Keywords:** Extracorporeal surgery, Giant ovarian cyst, Huge cystic pelvic mass, Multiport laparoscopy, Perioperative outcome, Transumbilical laparoendoscopic single-site

## Abstract

**Objective:**

To explore the clinical efficacy of transumbilical laparoendoscopic single-site extracorporeal surgery (TU-LESS-E) in the treatment of giant ovarian cysts and huge cystic pelvic masses.

**Methodology:**

This was a retrospective study. Sixty patients diagnosed with giant ovarian cysts and huge cystic pelvic masses undergoing laparoscopic ovarian cystectomy in Maternity & Child Care Center of Qinhuangdao from May 2022 to October 2024 were selected, and randomly divided into observation group and control group (n=30 each group). The control group was given conventional multiport laparoscopy, while the observation group was treated with TU-LESS-E cystectomy. Perioperative outcomes such as surgical duration, intraoperative blood loss and incidence of cystic fluid overflow, pain intensity six hours and 24 hours, one months and three months after surgery were compared between the two groups.

**Results:**

No significant differences were found in intraoperative blood loss or surgical duration between the two groups (*p>* 0.05). The incidence of cystic fluid overflow in the observation group was significantly lower than that in the control group (*p<* 0.05). The VAS score for incisional pain in the observation group was significantly lower than that in the control Group-6 and 24 hours after surgery (*p<* 0.05). The incidences of postoperative complications showed no significant differences between the two groups (*p>* 0.05).

**Conclusion:**

TU-LESS-E presents good results in the treatment of giant ovarian cysts and huge cystic pelvic masses. It can reduce surgical traumas and pain, the occurrence of cystic fluid overflow, as well as impact on ovarian reserve.

## INTRODUCTION

Benign ovarian cyst, also known as the cystic mass of the ovary, is one of the common diseases of the female genital organs^1^, and its occurrence may be influenced by various factors such as genetic factors, endocrine disorders and lifestyle. A cyst with a maximum diameter ≥ 10 cm on color Doppler ultrasound is usually considered a giant ovarian cyst. Surgical treatment is often recommended for ovarian cysts with obvious symptoms or uncertain nature, and it would be best for young female patients with surgical indications to choose lesion resection with ovarian function preservation.^2^ With the advantages of minimal traumas and rapid recovery, laparoscopic surgery is currently the preferred treatment for ovarian cysts.^3^

Nevertheless, traditional laparoscopy is difficult to operate, with significant limitations in imaging equipment and surgical instruments, as well as a high incidence of cystic fluid overflow. In addition, huge pelvic masses may obstruct the surgical field of view, and insufficient operating space will increase the incidence of collateral damage to adjacent organs during surgery. Consequently, its applicable population is limited. Furthermore, ovarian cysts with a diameter > 12 cm are more difficult to treat using laparoscopic surgery.^4^ As minimally invasive technique develops continuously, transumbilical laparoendoscopic single-site surgery is increasingly being applied in the diagnosis and treatment of gynecological diseases.^5^-^7^

Laparoendoscopic single-site extracorporeal surgery can achieve fine operations such as incising, peeling and suturing for lesions under direct vision and touch, which is conducive to reducing surgical difficulty and realizing smaller traumas and hidden surgical scars.^8^ In patients with giant ovarian cysts and huge cystic pelvic masses as subjects, this study intended to evaluate the clinical feasibility and safety of transumbilical laparoendoscopic single-site extracorporeal surgery (TU-LESS-E) in the treatment of giant ovarian cysts and huge cystic pelvic masses through comparing the clinical efficacy between TU-LESS-E and traditional multiport laparoscopy.

## METHODOLOGY

This was a retrospective study. Sixty patients diagnosed with giant ovarian cysts or huge cystic pelvic masses undergoing surgical treatment in Maternity & Child Care Center of Qinhuangdao from May 2022 to October 2024 were selected as subjects. Preoperatively, assessment of ovarian reserve was carried out in all patients. The patients were randomly divided into a control group (giant ovarian cyst: n=20, huge cystic pelvic mass: n=10) and an observation group (giant ovarian cyst: n=18, huge cystic pelvic mass: n=12), with 30 patients in each group.

### Ethical approval:

The study was approved by the Ethics Committee of Maternity & Child Care Center of Qinhuangdao (No.: QHDFY-20240902A01; date: September 02, 2024), and all patients signed an informed consent form.

### Inclusion criteria:

- Preoperative color Doppler ultrasound or pelvic and abdominal MRI suggested a cystic mass with a maximum diameter ≥ 10 cm, which was confirmed as giant ovarian cyst or huge cystic pelvic mass with surgical indications.
- Cysts were hypoechoic or anechoic, without solid components or papillary protrusions.
- Patients aged 20-40 years old with good communication skills.
- Family members were well informed of and agreed to the surgical plan.

### Exclusion criteria:

-Intolerance to laparoscopy or presence of surgical contraindications, such as a history of peritonitis, concomitant coagulation dysfunction, and severe cardiopulmonary diseases.
-With a history of retroperitoneal surgery or infectious disease.
-Severe pelvic adhesions.
-A previous history of malignant tumors or a family history of ovarian cancer.
-Suspected malignant tumors in intraoperative pathology.
-Pregnant women.
-Voluntary withdrawal in the midway.

### Preoperative preparation:

Routine preoperative examinations were performed, according to the function of giant ovarian cyst or huge cystic pelvic mass, the preparation of drugs before operation is generally 2-4 weeks, the blood pressure and heart rate are stable and the volume is expanded. The patients were fasted for six hour before surgery, and underwent routine preoperative skin and bowel preparation.

### Surgical methods:

The control group was given conventional multiport laparoscopy. After completing all preoperative examinations, the patients were punctured at the umbilical region, McBurney’s point, and midpoint between the anti-McBurney’s point and the navel, respectively, under general anesthesia and indwelling urinary catheterization. Next, Trocar was inserted and an artificial pneumoperitoneum was established. The entire pelvic and abdominal cavity was explored thoroughly, with intraoperative pneumoperitoneum pressure maintained at 12-14 mmHg. Later, the ovarian cortex on the affected side was dissected and bluntly separated from the cyst, and then removal of the ovarian cyst was performed using dissecting forceps combined with surgical scissors, with peritoneal lavage fluid retained for further cytological examination. Bleeding was stopped and the ovary was sutured by absorbable sutures. After surgery, the pelvic and abdominal cavity was rinsed with normal saline. With benign pathological results, the surgery was completed and the abdominal incision was sutured.

The observation group was treated with TU-LESS-E cystectomy. Preoperatively, the patients received indwelling urinary catheterization and general anesthesia. A 2.5-3-cm longitudinal incision was made at the center of the umbilical region. The abdominal cavity was entered by lifting both sides of the navel with tissue forceps, and dissecting the fascia and peritoneum successively at the midline of the umbilical region under direct vision. After fixing a protector at the incision site, the laparoendoscope was inserted via the incision, and an artificial pneumoperitoneum was established, with intraoperative pneumoperitoneum pressure maintained at 12-14 mmHg. It should be operated carefully to avoid overflow of cystic fluid when cutting open the tumor. It is difficult in obese patients to operate. After the establishment of retroperitoneal space, the fat should be removed carefully, the peritoneum and ovarian cortex should not be damaged.

The pelvic cavity, peritoneal surface, omentum, and ovary on both affected and contralateral sides were explored thoroughly, with peritoneal lavage fluid retained for next cytological examination. Adhesions were released using laparoscopic instruments. Under the laparoendoscope, the cyst was lifted to the incision at the umbilical region, and then the cystic wall was clamped with tissue forceps and lifted upwards, followed by a puncture between two pairs of tissue forceps and suction of cystic fluid. While suctioning the cystic fluid for volume reduction, the cyst was pulled out of the abdominal incision for ovarian cystectomy and ovarian suturing under direct vision. After the removal of ovarian cyst, the ovary was returned to the abdominal cavity. Subsequently, the pelvic cavity was examined carefully to exclude bleeding or other abnormal conditions, and then the pelvic and abdominal cavity was rinsed with normal saline and the fluid in the pelvic and abdominal cavity was fully suctioned. Finally, the laparoendoscope was withdrawn, and the peritoneum, fascia and subcutaneous tissue at the puncture site of the umbilical region were sutured successively. The maximum follow-up time for patients in both groups was three months, and case data collection ceased in October 2024.

### Evaluation indexes:

*Comparison of general clinical data:* General clinical data such as patient age, body mass index (BMI) and maximum mass diameter were compared between the two groups.

*Comparison of surgery-related indicators:* A statistical comparison was conducted on surgery-related indicators, such as intraoperative blood loss, surgical duration, and incidence of cystic fluid overflow, between the two groups.

*Comparison of postoperative pain:* The intensity of postoperative pain in both groups was assessed on a Visual Analogue Scale (VAS) 6 hoursours and 24 hours ours after surgery^9^, with a total score of 10. Pain intensity was positively correlated with the VAS score.

*Comparison of complications:* The incidences of postoperative complications, including incisional surgical site infection (SSI), postoperative blood loss, lower limb venous thrombosis, and collateral organ damage, were compared between the two groups.

*Ovarian reserve:* The levels of follicle-stimulating hormone (FSH), luteinizing hormone (LH), estradiol (E_2_) and antimullerian hormone (AMH) in both groups were measured before surgery, one month and three months after surgery, respectively.

### Statistical analysis:

The data were statistically analyzed using SPSS 27.0. Measurement and enumeration data were expressed as (`X ± s) and [n (%)], and analyzed by the *χ^2^* and *t* test, respectively. A *P* value < 0.05 was considered as statistically significant.

## RESULTS

Comparison of general clinical data The two groups showed no significant differences in general clinical data such as patient age, BMI, maximum mass diameter and history of pelvic surgery (*p>* 0.05) Table-I. No significant differences were found in intraoperative blood loss or surgical duration between the two groups (*p>* 0.05). Overflow of cystic fluid occurred in four patients of the control group, but not occurred in the observation group. The incidence of cystic fluid overflow in the observation group was significantly lower than that in the control group (*p<* 0.05) Table-II. The VAS scores for postoperative pain in both groups are displayed in Table-III. The results showed no significant differences in the incidence of postoperative complications between the two groups (*p>* 0.05) Table-IV. The measured levels of E_2_, LH, FSH and AMH in both groups before surgery, one month and three months after surgery are listed in Table-V.

**Table-I:** Comparison of general clinical data.

Index	Observation group (n = 30)	Control group (n = 30)	t/χ^2^	P
Age (year)	32.51 ± 2.62	31.97 ± 2.68	1.284	0.138
BMI (kg/m^2^)	23.16 ± 4.34	22.99 ± 4.62	0.705	0.092
History of pelvic surgery [n (%)]	2 (6.67)	3 (10.00)		0.451
Maximum mass diameter (cm)	16.72 ± 5.09	16.45 ± 4.86	1.169	0.512

**Table-II:** Comparison of surgery-related indicators.

Group	Intraoperative blood loss (ml)	Surgical duration (min)	Incidence of cystic fluid overflow
Observation group (n = 30)	42.36 ± 7.76	65.73 ± 7.05	0
Control group (n = 30)	44.12 ± 7.13	62.85 ± 7.37	4 (13.33)
*t/χ^2^*	1.695	0.913	
*P*	0.127	0.352	0.035

**Table-III:** Comparison of postoperative VAS score between the two groups (*χ̅*±*S*).

Group	Six hours after surgery	24 hours after surgery
Observation group (n = 30)	4.13 ± 1.09	1.87 ± 0.71^*^
Control group (n = 30)	4.96 ± 1.15	2.26 ± 0.93^*^
*t*	1.692	2.162
*P*	0.036	0.023

**Table-IV:** Comparison of postoperative complications.

Group	Incisional SSI	Collateral organ damage	Lower limb venous thrombosis	Postoperative blood loss	Total
Observation group (n = 30)	1(3.33)	0	1(3.33)	0	2(6.67)
Control group (n = 30)	1(3.33)	0	1(3.33)	0	3(10.00)
*t*					0.602
*P*					0.419

**Table-V:** Comparison of ovarian reserve between the two groups.

Group	E2(ng/ml)			LH(IU/L)		
	Before surgery	One month after surgery	Three months after surgery	Before surgery	One month after surgery	Three months after surgery
Observation group (n = 30)	166.53±11.03	134.27±13.34^*^	161.76±13.24^#^	7.27±2.05	12.37±2.36^*^	7.42±1.41^#^
Control group (n = 30)	164.47±10.77	116.14±12.57^*^	148.59±12.92^#^	7.15±1.89	12.79±2.47^*^	7.93±1.62^#^
*t*	1.915	6.754	6.328	1.306	7.538	6.153
*P*	0.373	0.001	0.001	0.466	0.001	0.04
***Group***	***FSH(U/ml)***			***AMH(ng/ml)***		
	***Before surgery***	***One month after surgery***	***Three months after surgery***	***Before surgery***	***One month after surgery***	***Three months after surgery***
Observation group (n = 30)	6.51±1.02	7.26±1.32^*^	6.73±1.25^#^	4.05±0.79	2.26±0.61^*^	3.53±0.71^#^
Control group (n = 30)	6.45±1.07	8.18±1.59^*^	7.02±1.32^#^	4.11±0.81	2.19±0.57^*^	2.80±0.69^#^
*t*	0.401	3.716	3.185	0.451	2.192	5.173
*P*	0.364	0.041	0.001	0.579	0.031	0.001

Compared with the same group before surgery,

* p< 0.05; compared with the same Group-1 month after surgery,

# p< 0.05.

## DISCUSSION

The present study showed no significant differences in intraoperative blood loss or surgical duration between the observation group and the control group (*p>* 0.05). The incidence of cystic fluid overflow in the observation group was significantly lower than that in the control group (*p<* 0.05). The intra-group comparison revealed that pain intensity 24 hours after surgery in both groups reduced significantly compared to that six hours after surgery (*p<* 0.05). The VAS score in the observation group was significantly lower than that in the control Group-6 and 24 hours after surgery (*p<* 0.05). The incidences of postoperative complications such as incisional SSI, intestinal adhesions and lower limb venous thrombosis showed no significant differences between the two groups (*p>* 0.05). These findings indicate that TU-LESS-E for giant ovarian cysts and huge cystic pelvic masses can reduce the risk of cystic fluid overflow and postoperative pain while ensuring surgical effectiveness.^10^,^11^ Ovarian reserve is an important index for evaluating reproductive function.^12^,^13^ Currently, the main indicators for the assessment of ovarian reserve in clinical practice include AMH, FSH, LH, E_2_, etc. AMH can regulate follicular development and participate in follicular growth^14^, and its serum level can effectively reflect ovarian reserve. When ovarian function declines, the AMH level will gradually decrease. It has been shown that when AMH < 1 ng/mL, the clinical pregnancy rate is generally low, and the pregnancy rate tends to increase as AMH increases.^15^ In addition, elevated FSH and LH, as well as reduced E_2_, all indicate a decline in ovarian reserve.^16^ Ovarian reserve can be declined due to endometriosis, chemotherapy and gynecological surgery, of which ovarian surgery is an important risk factor for causing a decline in ovarian reserve. Surgical treatment can cause partial loss of ovarian tissue to varying degrees, leading to hormonal imbalances in the body. Nevertheless, after cyst removal, ovarian function will gradually recover. Monitoring hormone levels before and after surgery can accurately predict the ovarian reserve of patients.^17^ Ovarian surgery is mostly performed in women of childbearing age. Therefore, understanding the impacts of different surgical methods on ovarian reserve is conducive to making decisions that are most beneficial for patients to preserve ovarian reserve, thereby reducing the occurrence of premature ovarian insufficiency (POI).

In the treatment of giant ovarian cysts, traditional laparoscopic surgery is restricted in its application because of the limited pelvic field of view, and a high risk of intraoperative extravasation of cystic fluid, which may lead to the spread of borderline or malignant tumors. Combining the advantages of traditional laparotomy and laparoscopic technique, TU-LESS-E is operated using the navel as a single approach, with a hidden incision, which can effectively overcome the shortcomings of traditional single-port laparoscopy and achieve fine operations for lesion resection under direct vision. Its therapeutic value in giant ovarian cysts is gradually being recognized. Zhang et al.^18^ have demonstrated that compared to traditional laparoscopy, TU-LESS-E has shorter surgical duration, and significantly reduced blood loss and cystic fluid overflow rate.

Our results showed that before surgery, ovarian reserve was not significantly different between the two groups. One and three months after surgery, E_2_ and AMH showed a first decreasing and then increasing trend, while LH and FSH presented a first increasing and then decreasing trend in both groups compared with those before surgery. The observation group exhibited lower FSH and LH levels while higher E_2_ and AMH levels compared with the control group one and three months after surgery, and the differences were statistically significant (*p<* 0.05), suggesting that both surgical procedures have a certain impact on ovarian reserve, and TU-LESS-E presents a smaller impact on the ovarian reserve of patients with giant ovarian cysts and huge cystic pelvic masses. According to the research results from Sharen et al.^19^, TU-LESS-E cystectomy for giant ovarian cysts and huge cystic pelvic masses can significantly reduce intraoperative bleeding loss, cystic fluid overflow rate and postoperative 24-h VAS score for incisional pain. In the study by Liang^20^, through comparing the safety and efficacy of transumbilical single-site laparoscopy and traditional laparoscopy in the treatment of benign gynecological tumors, it has been found that the former can reduce patients’ oxidative stress responses, help them recover ovarian function and shorten their hospital stay, but there are no significant differences in postoperative complications between the two methods.

### Limitations

However, this study also have shortcomings, such as a small number of samples, and this is a preliminary retrospective study. In future we will enlarge the number of patients and undertake long-term follow-up studies to improve the credibility of the conclusion and the level of evidence..

## CONCLUSION

TU-LESS-E adopts the navel as the surgical approach and is operated under direct vision, which can avoid cyst perforation, thus reducing surgical traumas, shortening postoperative recovery, and alleviating the negative impact of surgery on ovarian function of patients with giant ovarian cysts and huge cystic pelvic masses. Therefore, it is worth promoting.

### Authors’ Contributions:

**YH** and **YW:** Performed the studies, participated in collecting data, drafted the manuscript, and are responsible and accountable for the accuracy or integrity of the work.

**BS** and **R:** Study design, Statistical analysis, Critical review.

**LG: A**cquisition, analysis, or interpretation of data and drafted the manuscript.

All authors have read and approved the final manuscript.
